# Examining the Corn Seedling Emergence–Temperature Relationship for Recent Hybrids: Insights from Experimental Studies

**DOI:** 10.3390/plants12213699

**Published:** 2023-10-27

**Authors:** Sahila Beegum, Charles Hunt Walne, Krishna N. Reddy, Vangimalla Reddy, Kambham Raja Reddy

**Affiliations:** 1 Adaptive Cropping Systems Laboratory, United States Department of Agriculture, Agricultural Research Service, Beltsville, MD 20705, USA; 2 Nebraska Water Center, Robert B. Daugherty Water for Food Global Institute, 2021 Transformation Drive, University of Nebraska, Lincoln, NE 68588, USA; 3 Department of Plant and Soil Sciences, Mississippi State University, Starkville, MS 39762, USA; 4 United States Department of Agriculture, Agricultural Research Service, Crop Production Systems Research Unit, 141 Experiment Station Road, P.O. Box 350, Stoneville, MS 38776, USA

**Keywords:** corn seedling emergence, growing degree days, hybrids, SPAR, functional relationship, base temperature

## Abstract

Corn seedling emergence is a critical factor affecting crop yields. Accurately predicting emergence is crucial for precise crop growth and development simulation in process-based crop models. While various experimental studies have investigated the relationship between corn seedling emergence and temperature, there remains a scarcity of studies focused on newer corn hybrids. In the present study, statistical models (linear and quadratic functional relationships) are developed based on the seedling emergence of ten current corn hybrids, considering soil and air temperatures as influencing factors. The data used for model development are obtained from controlled soil plant atmospheric research chamber experiments focused on corn seedling emergence at five different temperatures. Upon evaluating the developed models, the quadratic model relating the air temperature with time to emergence was found more accurate for all corn hybrids (coefficient of determination (R^2^): 0.97, root mean square error (RMSE): 0.42 day) followed by the quadratic model based on soil temperature (R^2^: 0.96, RMSE: 1.42 days), linear model based on air (R^2^: 0.94, RMSE: 0.53 day) and soil temperature (R^2^: 0.94, RMSE: 0.70 day). A growing degree day (GDD)-based model was also developed for the newer hybrids. When comparing the developed GDD-based model with the existing GDD models (based on old hybrids), it was observed that the GDD required for emergence was 16% higher than the GDD used in the current models. This showed that the existing GDD-based models need to be revisited when adopted for newer hybrids and adapted to corn crop simulation models. The developed seedling emergence model, integrated into a process-based corn crop simulation model, can benefit farmers and researchers in corn crop management. It can aid in optimizing planting schedules, supporting management decisions, and predicting corn crop growth, development, and it yields more accurately.

## 1. Introduction

Corn seedling emergence is a crucial factor that significantly affects the final yield of this widely grown crop [1]. A delay in the emergence by a few hours can shift the critical growth stages to a different environment and influence crop yield [2,3]. The corn seedling emergence model is vital in process-based crop models because it represents the primary and initial stages of crop growth. Crop models are found to be sensitive to errors associated with the inaccurate simulation of the seedling emergence [4]. Therefore, it is essential to model this process correctly to ensure the accurate simulation of subsequent plant growth and development. A more precise seedling emergence model enables farmers to adjust planting schedules and anticipate the optimal field operation times.

A range of factors influences corn seedling emergence: temperature, soil aeration, soil matric potential, seeding depth, seed quality, seed size, soil aggregate size distribution, soil type, and management practices (e.g., mulch, tillage, planting the seed directly into cover crops) and biotic factors (pest and diseases) [5,6,7]. Temperature is one of the most critical factors affecting seedling emergence [8,9]. A significant reduction in the rate and percentage of corn seedling emergence is observed at lower temperatures below 10 °C [7,10,11]. At lower temperatures, the seeds absorb water but do not initiate root or shoot growth, which leads to seed rot and poor emergence [12]. The optimum temperature for corn seedling emergence is observed to be between 20 and 30 °C, as reported in several studies [10,13,14,15]. Early planting is sometimes adopted for corn to avoid excessive heat/drought that may impact grain production during its reproductive phase [12,16,17]. However, early planting may be influenced by the wet soil conditions and cooler temperatures at the planting time, especially during the seedling emergence [18,19]. Gupta, 1985 observed that this is more prominent in the northern corn belt than in the southern corn belt in the USA [20].

Several studies on corn seedling emergence and temperature relationships [1,5,7,8,21]. Most of these studies are based on old corn hybrids (XIA5 and W346, 1983, SMASV2, DeKalb Pfizer T 1000) (Table 1). Most of the growing degree day-based models (GDD models) currently used to predict corn seedling emergence are developed based on these studies that examined older corn hybrids [8]. Newer corn hybrids are often developed with various characteristics to cater to different agricultural needs and challenges [22,23,24]. These hybrids may have different temperature requirements for germination and emergence than traditional varieties/hybrids.

Another aspect to consider is whether soil or air temperature should be correlated with emergence. A few studies have attempted to establish a correlation between seedling emergence and the combined effects of soil and air temperature [10]. Certain studies have demonstrated that soil temperature correlates more accurately with seedling emergence than air temperature [7,8,13]. Most of these studies have investigated the relationship with air temperature alone, primarily due to the complexity and unavailability of soil temperature measurements.

Developing a soil/air temperature-based emergence model that accounts for the specific temperature requirements of recent corn hybrids is essential. Such a model would help corn producers anticipate the optimal planting times and conditions for particular hybrids, increasing the likelihood of successful emergence. Accurate emergence prediction can also help in resource management and yield prediction.

The present study aims to develop a temperature-based corn seedling emergence model using data from soil plant atmospheric research (SPAR) chamber experiments, explicitly focusing on newer corn hybrids. The specific objectives of this study are to (a) identify the recent corn hybrids that are currently grown in the USA, (b) carry out SPAR chamber experiments under different temperature controls for the selected corn hybrids, (c) develop corn seedling emergence models based on measured time to seedling emergence, air and soil temperature, (d) evaluate the developed models using a different set of measured corn emergence data collected from SPAR experiments and (e) compare the developed model with existing corn seedling emergence models and assess its accuracy and practical usefulness. The study focuses on utilizing SPAR chambers to facilitate controlled temperature conditions across a range of temperatures that may not be feasible in field-scale experiments.

The present study hypothesizes that soil and air temperatures significantly influence seedling emergence. Furthermore, there could be variations in the emergence rate when comparing simulations using existing models with the models developed in this study for newer hybrids.

## 2. Materials and Methods

### 2.1. Selection of Recent Corn Hybrids

Ten recent corn hybrids were selected to represent the current corn hybrids generally grown in the USA [29]. Table 2 offers the list of hybrids, with companies and the names of the hybrids. The hybrids were obtained from Agrigold, Augusta, Dekalb, Progeny, and Croplan, Dynagro companies.

### 2.2. Experiment Facilities and Setup

Experiments were conducted to measure the seedling emergence of ten recent corn hybrids (Table 2) at five different day/night temperatures. The experiments were conducted in sunlit SPAR units at the Rodney Soil Plant Science Research Center, Mississippi State University, Mississippi [30]. Five SPAR units were used for the experiments [30]. Each of the units was set to a day/night air temperature treatment of 18/12 °C, 22/16 °C, 26/20 °C, 30/24 °C, and 34/28 °C and an average carbon dioxide (CO_2_) concentration of 420 ppm (ambient CO_2_ concentration). Each SPAR unit had ten plastic pots (15.2 cm diameter and 30.5 cm height) for ten hybrids, with a 0.5 cm diameter hole at the bottom for excess water and nutrient drainage. Each pot contained 0.5 kg of gravel at the bottom, and the rest was filled with pure sand (particle size less than 0.3 mm). The plants were irrigated and fertilized with full-strength Hoagland nutrient solution using an automated drip irrigation system [31]. A total of 40 seeds (4 seeds per hybrid in each of the ten pots) were seeded at a depth of 2.5 cm in each of the five SPAR units, resulting in 50 pots and 200 seeds.

### 2.3. Measurements

To represent the seedling emergence, ‘Time to 50% seedling emergence (T50%)’ was used as it provides a more practical estimate of typical emergence time [4]. The T50% was recorded when the coleoptile and first leaves emerged through the soil surface [32].

Soil and air temperatures were measured for each temperature treatment in this study. In the SPAR chambers, the air temperatures were monitored using aspirated, shielded thermocouples. For measuring the soil temperature, the thermocouples were inserted at a 5 cm depth from the soil surface [30].

### 2.4. Data Analysis

First, the variation in the T50% among all ten hybrids under five different day/night temperature treatments was analyzed. Mean, standard deviation, and two-way analysis of variance (ANOVA) were analyzed among the hybrids. To identify statistically significant differences between treatment groups, Fisher’s LSD test was performed. This test is suitable for conducting pairwise comparisons following ANOVA due to its efficiency and sensitivity, making it well-suited for detecting subtle distinctions in experimental data.

This was followed by developing the functional relationship (linear and non-linear or quadratic) between T50% and the soil and air temperature. The developed models were then compared with the existing Growing Degree Days (GDD)-based models for corn seedling emergence. In GDD-based models, the GDD accumulates by subtracting the base temperature (T_base_: temperature above which the developmental processes occur) from the average daily temperature and summing these differences over time [25]. The developed model’s goodness of fit, accuracy, and practical usefulness were evaluated using metrics: coefficient of determination (R^2^), root means square error (RMSE), standard deviation (SD), standard error (SE), and envelope of acceptable precision (EAP) [33].

R^2^ is a statistical measure that determines how well the regression line fits the observed data. A value of 0 indicates that the regression line does not fit the data at all, while a value of 1 indicates a perfect fit. The SD is a measure of the amount of variability in a dataset. The SE measures the accuracy with which a sample distribution represents a population using standard deviation. RMSE is estimated as the square root of the mean squared error. The lower the value of the RMSE, the better the model. The EAP is a range of values defining the acceptable error level in a predictive model. It is used to determine whether the model is accurate enough to be used in decision making. This is estimated by analyzing the percentage of the deviation (predicted–measured) that falls within a predetermined percentage of allowable error relative to the measured values [33].

## 3. Results

### 3.1. Data Analysis and Model Development

**Figure 1:**
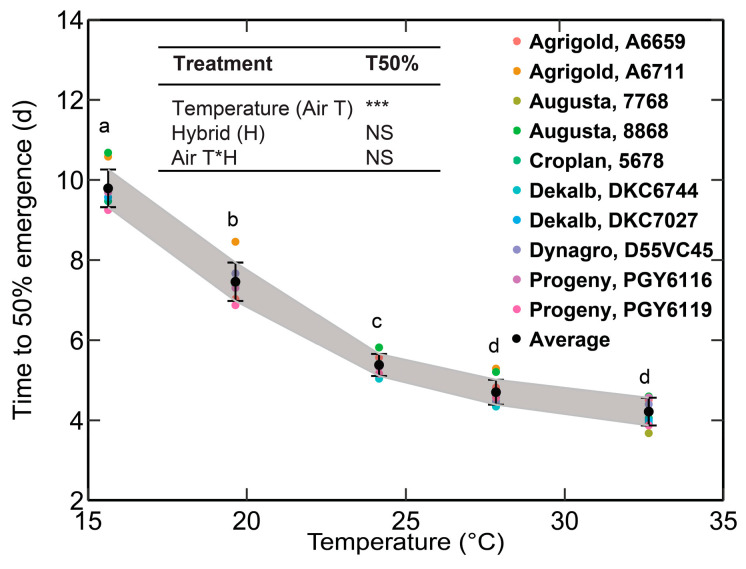
Time to 50% emergence (T50%) for each hybrid, average value, and the standard deviation for the five temperature treatments. A summary of the two-way analysis of variance across hybrids and temperature is added in Figure 1. Significant variations are indicated by *** (*p* < 0.001) and non-significant; NS (*p* > 0.05) (Fisher’s LSD test). Different lowercase letters (e.g., a, b, c, d) indicate significant differences in T50% at *p* < 0.05. T50% sharing the same lowercase letters shows they are not significantly different.

Table 2 presents the details of the T50% measured from the SPAR experiments. Only a minor variation was observed in T50% among the ten corn hybrids (maximum SD of 0.47 day and average SD of 0.37 day) (Figure 1). Based on the Fisher’s LSD test, the variations in the T50% among the hybrids were insignificant (*p* > 0.05). Therefore, the average T50% values among the hybrids were used for further analysis and model development. The average T50% was observed to be decreasing with an increase in temperature. The average T50% decreased from 9.79 days to 4.22 days when the temperature was increased from 18/12 °C to 34/28 °C (Figure 1, Table 2).

**Figure 2:**
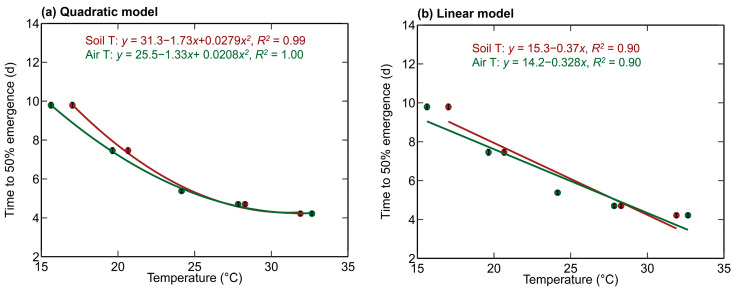
Quadratic model (**a**) and linear model (**b**) offer the functional relationships between the temperature (air and soil) and T50%. The mean ± standard error of four replications for each hybrid is also presented. R^2^ is the coefficient of determination, ‘Soil T’ is the soil temperature, and ‘Air T’ is the air temperature.

Based on the measured average T50%, functional relationships between temperature and T50% were obtained by fitting a quadratic model (Figure 2a) and a linear model (Figure 2b) as a function of air and soil temperature. The R^2^ of all the developed models was greater than 0.9. Among all four models, the quadratic model as a function of air temperature resulted in the highest R^2^ equal to 1.0.

### 3.2. Model Evaluation

**Figure 3:**
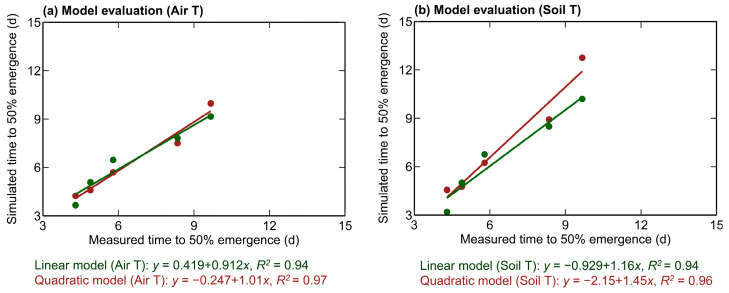
Simulated and measured time to 50% emergence based on linear and quadratic models, which is a function of air temperature (**a**) and soil temperature (**b**). R^2^ is the coefficient of determination, ‘Soil T’ is the soil temperature, and ‘Air T’ is the air temperature. The linear model (Air T) and quadratic model (Air T) represent the linear and quadratic models as a function of air temperature. Linear model (Soil T) and quadratic model (Soil T) represent the linear and quadratic models as a function of soil temperature.

Another set of experiments was carried out in controlled SPAR chambers using a similar methodology discussed in Section 2.2 to evaluate the developed linear and quadratic model. These experiments were conducted for ten different recent corn hybrids (listed in Table 2), subjected to varying day/night temperature treatments of 18/12 °C, 22/16 °C, 26/20 °C, 30/24 °C, and 34/28 °C. The T50% values obtained from the evaluation experiments were compared with the model-simulated T50% for different temperatures (Figure 3a,b).

The quadratic and linear models simulated the T50% values with an R^2^ value greater than 0.94. The developed quadratic model relating the air temperature with T50% was more accurate for all corn hybrids (R^2^: 0.97, RMSE: 0.42 day), followed by the quadratic model based on soil temperature (R^2^: 0.96, RMSE: 1.42 days), a linear model based on air (R^2^: 0.94, RMSE: 0.53 day) and soil temperature (R^2^: 0.94, RMSE: 0.70 day) (Figure 3a,b). This indicates that the developed linear and quadratic models can reasonably simulate T50% with higher accuracy in the simulations when using the quadratic models.

## 4. Discussion

### 4.1. Decrease in T50% with Increase in Temperature

From the current study, the average T50% was observed to be decreasing with an increase in the temperature (Section 3.1, Figure 1, Table 2). A similar observation was made by Alessi et al. (1971) [8]. Beauchamp and Lathwell, 1967 and Willis et al., 1957 observed an increase in time to seedling emergence from 4 days at 25 °C to 16 days at 12.5 °C [13,34]. Adams, 1967 observed an increase in time to emergence from 5 to 13 days with a decrease in temperature from 21 to 13 °C [35]. Lower soil temperature affects seed vigor, lowers metabolic processes and root development, and delays seed emergence [36]. The energy within the seed needed to push the coleoptile out of the ground is also depleted at lower temperatures.

### 4.2. Quadratic Function Better Represented the Variation in the T50% with Temperature

Based on the functional relationships developed between T50% and temperature, it was observed that the quadratic relationship better represented the variation compared to the linear function. A similar observation was made by Warrington and Kanemasu, 1983, and Edalat and Kazemeini, 2014 who observed that the relationship between seedling emergence and temperature is non-linear or curvilinear [15,21].

### 4.3. Soil Temperature and Air Temperature

Although both soil temperature and air temperature affect seed emergence and can be used to estimate seed emergence, the soil temperature strongly influences the seed emergence rate [1,10]. Due to challenges associated with measuring soil temperature, most of the existing models for corn emergence are based on air temperature rather than soil temperature. There were also attempts to establish a relationship between air and soil temperature and to utilize the soil temperature corresponding to the air temperature to estimate corn seedling emergence [10]. In the present study, when comparing soil and air temperatures, it was observed that the soil temperature is higher (+1.3 °C) at a lower air temperature (18/12 °C) and lower (−0.75 °C) at a higher air temperature (30/24 °C). Establishing a direct relationship between soil and air temperature is challenging, as multiple factors (solar radiation, soil water content, soil texture, elevation, slope, aspect, biogeochemistry, etc.) influence it [37].

### 4.4. Optimum Temperature for Seedling Emergence

Based on the developed quadratic models, the optimum temperature (with minimum time to emergence) estimated from the current study was 31.97 °C for air temperature and 28.82 °C for soil temperature. These values are similar to the optimum temperature ranges reported in the previous literature: 30 °C [21], 25 °C to 35 °C [8], 28 °C to 30 °C [15], and 20 °C to 30 °C [1] (Table 1).

### 4.5. Comparison of the Developed Models with the Existing GDD-Based Models

The developed linear and quadratic models are subsequently compared with the GDD-based models. Two distinct GDD-based models are taken into consideration. The first model is based on existing GDD-based models sourced from the literature, which are currently utilized for simulating corn seedling emergence. The second model is a GDD model specifically developed for the newer hybrids from the present study. Since the GDD-based models are generally a function of air temperature (and not soil temperature), the comparison is carried out between the linear and quadratic models as a function of air temperature with the GDD-based models.

The reported values of GDD for T50% from the previous studies for a 10 °C T_base_ are 58 [1], 58.56 [14], 69 [38], 80 [39], 58 [10], 69 GDD [7] (Table 1). Based on this, an average value of 65 GDD with a 10 °C T_base_ is considered as the GDD model from the literature. This GDD model is represented as ‘GDD65/T_base_10 °C-Literature’ hereafter. A GDD model is developed from the current study by assuming the T_base_ in the current study as 10 °C (similar to that observed in the literature) (Table 1). This GDD model developed in the current study is referred to as ‘GDD76/T_base_10 °C-Present’ hereafter. From the present study, the GDD at 10 °C T_base_ is observed to be 76 GDD. This shows that the recent hybrids require a 16% higher GDD (76) compared to the 65 GDD for the older hybrids for T50%.

A different GDD requirement for the newer hybrids compared to older hybrids could be due to genotype–environmental interactions [40]. It might be possible that newer corn hybrids have been genetically improved for other traits, such as disease resistance, drought tolerance, or yield potential, which could inadvertently affect their GDD requirements for seedling emergence [41]. The increased GDD requirement in newer hybrids could reflect such trade-offs, hinting at complex interactions between genetic, physiological, and environmental factors [42].

**Figure 4:**
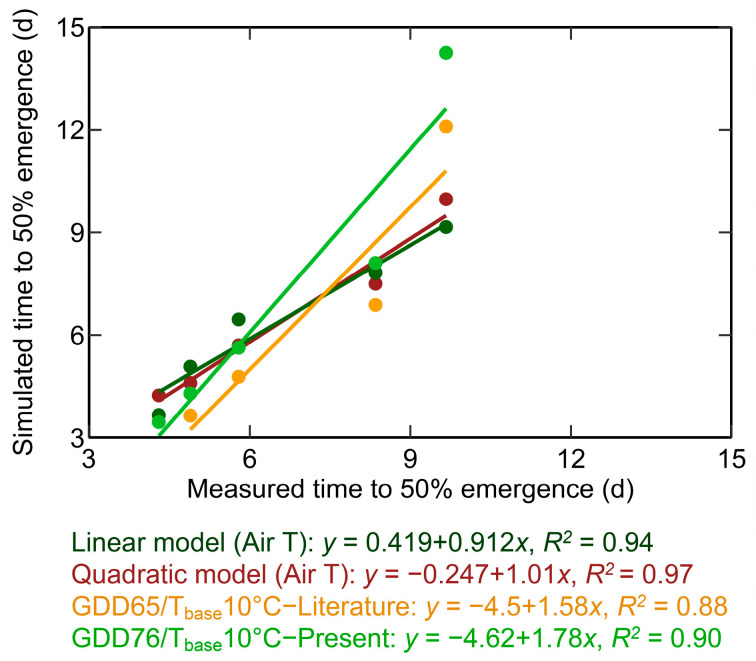
Simulated and measured time to 50% emergence based on linear and quadratic models and GDD-based models, which is a function of air temperature. The linear model (Air T) and quadratic model (Air T) represent the linear and quadratic models as a function of air temperature. ‘GDD65/T_base_10 °C—literature’ refers to the existing GDD-based corn seedling emergence model with 65 GDD and a base temperature (T_base_) of 10 °C. ‘GDD76/T_base_10 °C—present’ (76 GDD and 10 °C T_base_) represents the GDD-based model developed in the present study.

The measured and simulated T50% using the linear and quadratic model as a function of air temperature, GDD65/T_base_10 °C—literature, and GDD76/T_base_10 °C—present, are presented in Figure 4. Among the GDD models, GDD76/T_base_10 °C—present (R^2^: 0.90, RMSE: 2.1 days) better predicted the T50% than GDD65/T_base_10 °C—literature (R^2^: 0.88, RMSE: 1.57 days) (Figure 4). GDD65/T_base_10 °C—literature and GDD76/T_base_10 °C—resent underpredicted the T50% at higher temperatures and overpredicted at lower temperatures (Figure 4).

**Figure 5:**
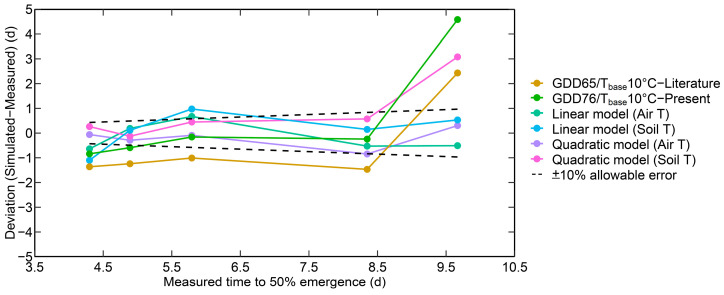
Deviation between the model simulated and measured time to 50% emergence for different corn seedling emergence models. ‘Soil T’ is the soil temperature, and ‘Air T’ is the air temperature. The linear model (Air T) and quadratic model (Air T) represent the linear and quadratic models as a function of air temperature. The linear model (Soil T) and quadratic model (Soil T) represent the linear and quadratic models as a function of soil temperature. ‘GDD65/T_base_10 °C—literature’ refers to the existing GDD-based corn seedling emergence model with 65 GDD and a base temperature (T_base_) of 10 °C. ‘GDD76/T_base_10 °C—present’ (76 GDD and 10 °C T_base_) represents the GDD-based model developed in the present study.

The performance of the developed linear and quadratic models based on air and soil temperature, along with GDD-based corn seedling emergence models (from past studies/literature), is collectively analyzed and presented in Figure 5. Figure 5 shows the deviation (difference between simulated and measured) among different models to the predetermined percentage (10% EAP) of the allowable error relative to the measured T50% [33]. The order of model performance is as follows: quadratic model as a function of air temperature (EAP: 100%, R^2^: 0.97), quadratic model based on soil temperature (EAP: 80%, R^2^: 0.96), linear model as a function of air temperature (EAP: 60%, R^2^: 0.94), linear model as a function of soil temperature (EAP: 60%, R^2^: 0.94), newly developed GDD-based model with 10 °C T_base_ and 76 GDD (EAP: 40%, R^2^: 0.90), existing GDD-based model with 10 °C T_base_ and 76 GDD (EAP: 0%, R^2^: 0.88).

The percentage of simulated values within the 10% EAP was 100%, 80%, 60%, and 60% for the quadratic model (air temperature), quadratic model (soil temperature), linear model (air temperature), and linear model (soil temperature), respectively. The linear models were more accurate at low temperatures (towards higher T50% in Figure 5) than at high temperatures (towards lower T50%), and quadratic models behave conversely (Figure 5). The percentages of T50% simulated using GDD65/T_base_10 °C—literature and GDD76/T_base_ 10 °C—resent within 10% EAP were 0% and 40%, respectively. Among the GDD models, the GDD76/T T_base_ 10 °C—present was more efficient than GDD65/Tbase10 °C—literature (Figure 5). This shows that the existing GDD model (GDD65/T_base_10 °C—literature) needs to be revisited when adopted for newer hybrids and adapted to corn crop simulation models.

Since new hybrids are shown to require a 16% higher GDD based on this study, growers may need to plant corn earlier to ensure the necessary growing degree days for emergence. Delayed planting may increase the risk of exposure to adverse weather conditions. An extended time to emergence may result in a longer growing season for the crop, which can be advantageous in areas with favorable climates, allowing for increased vegetative and reproductive growth. If adjusting management practices is not feasible, considering the longer GDD requirements for newer hybrids, growers need to reconsider the selection of corn hybrids.

### 4.6. Limitations and Future Scope of the Study

The current study examined ways in which air and soil temperature affect the emergence of newer hybrid corn seeds. However, since new hybrids are consistently being developed and introduced through cross-breeding, it is important to note that while the model developed in this study can provide a foundational understanding, it may not be universally applicable to all forthcoming hybrid variations. The study only examined temperature’s impact on seed emergence; other factors, such as seed depth, soil properties, and seed quality, etc., were not considered. Future studies should analyze the effects of these factors on the seedling emergence of newer hybrids. In the present study, the T_base_ for the recent hybrids was assumed to be the same as in the literature (10 °C). A more precise value of the T_base_ for the current hybrids can be obtained by conducting experiments at lower temperature ranges (less than 18/12 °C day/night temperature). The emergence model was evaluated using SPAR chamber experiments with the same soil and temperature conditions used for model development. The model was not tested on different soil types, field-scale data, or different temperature conditions for corn seedling emergence. Including such data would increase the robustness and applicability of the findings. The developed model can be incorporated into a process-based corn crop model followed by model evaluation.

## 5. Summary and Conclusions

The study’s main objective was to examine the correlation between the corn seedling emergence and air/soil temperature for newer corn hybrids and compare the developed relationship with the existing GDD-based corn emergence model. In alignment with this objective, the present study developed corn seedling emergence models using data from SPAR chamber experiments under five different temperature ranges for ten recent corn hybrids. Only a minimal variation was observed in the T50% at each temperature among the new hybrids. The developed models (quadratic/linear and GDD-based models) for the recent hybrids and the existing GDD-based models were evaluated based on their fit, accuracy, and practical usefulness. The study results showed that the developed quadratic/linear and GDD-based models performed better than the existing GDD models. This indicates that the current GDD models need to be revisited when adopted for newer hybrids and adapted to corn crop simulation models. Once the newly developed emergence model is integrated into a process-based corn crop model, it can provide helpful insights for producers and researchers to adjust planting schedules according to environmental conditions and enhance the accuracy of corn crop growth and development simulations.

## Figures and Tables

**Table 1:** Previous experimental studies on corn seedling emergence. The base temperature (T_base_: temperature above which the developmental processes occur), growing degree days (GDD) required for emergence, the optimum temperature for corn seedling emergence, and the hybrids used in each experimental study are presented here. In GDD-based models, the accumulated GDD is obtained by subtracting the T_base_ from the average daily temperature and summing these differences over time [25]. Relevant information, when not available in the study, is marked as ‘No information’.

Experimental Study	T_base_	GDD	Optimum Temperature	Hybrids Used
[8]	10 °C	68 GDD for 80% emergence	25 to 35 °C	No information
[10]	10 °C	58 GDD for 50% emergence71 GDD for 75% emergence	No information	PrideK730, FunksG-4083, 391133R, A632XW117, W64AXCM105, M017XB73
[1]	10 °C	40 GDD to begin emergence and 60 GDD for 75% emergence	20 to 30 °C	No information
[26]	10 °C	80 GDD for 75% emergence	No information	No information
[13]	10 °C	No information	33.8 °C	No information
[21]	9 °C	62.5 GDD for 50% emergence	30 °C	XIA5 and W346
[14]	10 °C	52 GDD for 50% emergence and 62.5 GDD for 75% emergence	No information	Pioneer 3902
[15]	9.4 to 9.9 °C	No information	28.9 to 30.0 °C	SC704, BC666 and ZP677
[27]	8 °C	No information	34 °C	Pacific Hycorn42, DeKalb DK529, De Kalb XL82, Pacific Hycorn 83
[7]	10 °C	45 to 55 GDD to begin emergence and 70 to 80 GDD for 100% emergence	Temperature greater than 25 °C	DeKalb Pfizer T 1000
[28]	10 °C	64 to 86 GDD for 80% emergence	No information	No information
[20]	10 °C	59 to 76 GDD for 75% emergence	No information	No information

**Table 2:** Measured time to 50% emergence in all the hybrids at different day/night temperatures. Analysis of variance across hybrids on time to 50% emergence at different temperatures and the variance across temperatures for the average time to 50% emergence (averaged for all hybrids) are presented. Lowercase letters denote the statistical difference between the time to 50% emergence of the hybrids at each temperature treatment and between the time to 50% emergence (averaged among hybrids) and temperature treatments. Significant variations are indicated by *** (*p* < 0.001) and non-significant; NS (*p* > 0.05) (Fisher’s LSD test). Temperature*** represents a significant variation in the average time to 50% emergence at different temperatures.

			Air Temperature (°C) (Day/Night)				
			18/12 °C	22/16 °C	26/20 °C	30/24 °C	34/28 °C
			Time to 50% Emergence (Days)				
Sl. No.	Company	Hybrid Name	NS	NS	NS	NS	NS
1	Agrigold	A6659	9.47 a	7.03 a	5.57 a	4.82 a	4.51 a
2	Agrigold	A6711	10.58 a	8.46 a	5.82 a	5.29 a	4.57 a
3	Augusta	7768	9.69 a	7.67 a	5.17 a	4.57 a	3.68 a
4	Augusta	8868	10.68 a	7.39 a	5.82 a	5.21 a	4.60 a
5	Dekalb	DKC6744	9.81 a	7.67 a	5.04 a	4.35 a	3.93 a
6	Progeny	PGY6119	9.25 a	6.88 a	5.17 a	4.57 a	3.88 a
7	Croplan	5678	9.47 a	6.88 a	5.42 a	4.57 a	4.06 a
8	Dekalb	DKC7027	9.57 a	7.67 a	5.33 a	4.62 a	4.00 a
9	Progeny	PGY6116	9.69 a	7.31 a	5.33 a	4.57 a	4.57 a
10	Dynagro	D55VC45	9.69 a	7.67 a	5.17 a	4.47 a	4.40 a
Temperature***			18/12 °C	22/16 °C	26/20 °C	30/24 °C	34/28 °C
Average time to 50% emergence among hybrids							
			9.79 a	7.46 b	5.38 c	4.70 d	4.22 d

## Data Availability

Data are included in the manuscript.
